# Supplementary material for: Integrated relationship of nasopharyngeal airway host response and microbiome associates with bronchiolitis severity
Source: Nat Commun. 2022 Aug 30;13:4970. doi: 10.1038/s41467-022-32323-y (PMC9427849; doi:10.1038/s41467-022-32323-y)
Supplement: Supplementary file 1 — Supplementary Information [file 41467_2022_32323_MOESM1_ESM.pdf]

SUPPLEMENTARY INFORMATION

CONTENTS

Supplemental Table 1 Principal investigators at the 17 participating sites in MARC-35……………………….….3

Supplemental Table 2. Comparisons between analytic and non-analytic cohorts in the MARC-35……………..4

Supplemental Table 3. Pathway enrichment analysis of each module of weighted gene co-expression network analysis in host response…………………………………………………………………….……………………6

Supplemental Table 4. Assigned microbial species of each module of weighted gene co-expression network analysis in nasopharyngeal airway microbial composition………………………………….…………………..13

Supplemental Table 5. Pathway enrichment analysis of each module of weighted gene co-expression network analysis in nasopharyngeal airway microbial function…………………………….……………………....17

Supplemental Table 6. Primers for respiratory pathogen testing...…………………….……………………....21

Supplemental Figure 1. Study flow diagram …………………………………….…………….………...23

Supplemental Figure 2. Gene set enrichment analysis of host transcriptome data with regard to the use of positive pressure ventilation in infants hospitalized for bronchiolitis………………….…………….……….25

Supplemental Figure 3. Relationship of abundant fungal species with the risk of higher severity in infants hospitalized for bronchiolitis…………………...……………………………………………………….….27

Supplemental Figure 4. Gene set enrichment analysis of microbial function data with regard to the use of positive pressure ventilation in infants hospitalized for bronchiolitis…………………...……………………29

Supplemental Figure 5. Sensitivity analysis: Integrated relationship of the dual-transcriptome modules with the risk of intensive care use in infants hospitalized for bronchiolitis………………………….........………...……31

Supplemental Figure 6. Sensitivity analysis adjusting for age, sex, race/ethnicity, and virus: Integrated relationship of the dual-transcriptome modules with the risk of positive pressure ventilation use in infants hospitalized for bronchiolitis………………………………………………………...…………….….….……32

Supplemental Figure 7. Sensitivity analysis adjusting for age, sex, race/ethnicity, and virus: Integrated relationship of the dual-transcriptome modules with the risk of intensive care use in infants hospitalized for bronchiolitis……………………………………………………………………..………………………33

Supplemental Figure 8. Correlation network of major clinical variables and dual-transcriptome modules………………………………………………………………………………………….………34

Supplemental Figure 9. Log-log plot of whole-network connectivity distribution of host response, microbial composition, and microbial function…………………………………………...…….….……………………36

Supplemental Table 1. Principal investigators at the 17 participating sites in MARC-35

Amy D. Thompson, MD	Alfred I. duPont Hospital for Children, Wilmington, DE
Federico R. Laham, MD, MS	Arnold Palmer Hospital for Children, Orlando, FL
Jonathan M. Mansbach, MD, MPH	Boston Children's Hospital, Boston, MA
Vincent J. Wang, MD, MHA and Susan Wu, MD	Children's Hospital of Los Angeles, Los Angeles, CA
Michelle B. Dunn, MD and Jonathan M. Spergel, MD, PhD	Children's Hospital of Philadelphia, Philadelphia, PA
Juan C. Celedón, MD, DrPH	Children's Hospital of Pittsburgh, Pittsburgh, PA
Michael R. Gomez, MD, MS-HCA and Nancy Inhofe, MD	The Children's Hospital at St. Francis, Tulsa, OK
Brian M. Pate, MD and Henry T. Puls, MD	The Children's Mercy Hospital & Clinics, Kansas City, MO
Stephen J. Teach, MD, MPH	Children's National Medical Center, Washington, D.C.
Richard T. Strait, MD and Stephen C. Porter, MD, MSc, MPH	Cincinnati Children's Hospital and Medical Center, Cincinnati, OH
Ilana Y. Waynik, MD	Connecticut Children's Medical Center, Hartford, CT
Sujit Iyer, MD	Dell Children's Medical Center of Central Texas, Austin, TX
Michelle D. Stevenson, MD, MS	Norton Children's Hospital, Louisville, KY
Wayne G. Shreffler, MD, PhD and Ari R. Cohen, MD	Massachusetts General Hospital, Boston, MA
Anne K. Beasley, MD and Cindy S. Bauer, MD	Phoenix Children's Hospital, Phoenix, AZ
Thida Ong, MD and Markus Boos, MD, PhD	Seattle Children's Hospital, Seattle, WA
Charles G. Macias, MD, MPH	Texas Children's Hospital, Houston, TX

Supplemental Table 2. Comparisons between analytic and non-analytic cohorts in the MARC-35 cohort

Variables	Analytic cohort (n=244)	Non-analytic cohort (n=772)	P-value
**Baseline characteristics**			
Age (month), median (IQR)	3 (2-6)	3 (2-6)	0.75
Female sex	98 (40)	308 (40)	0.99
Race/ethnicity			0.99
Non-Hispanic white	102 (42)	328 (42)	
Non-Hispanic black	57 (23)	182 (24)	
Hispanic	76 (31)	232 (30)	
Others	9 (4)	30 (4)	
Parental history of asthma	76 (31)	269 (35)	0.31
Maternal smoking during pregnancy	34 (14)	113 (15)	0.86
C-section delivery	84 (34)	264 (34)	0.99
Prematurity (32-36.9 weeks of gestation)	47 (19)	139 (18)	0.73
Mostly breastfed for the first 3 month of age	115 (47)	357 (46)	0.48
Ever attended daycare	71 (29)	163 (21)	0.01
Previous breathing problems before the index hospitalization			0.21
1 episode	30 (12)	130 (17)	
> 2 episodes	10 (4)	36 (5)	
History of eczema	31 (13)	118 (15)	0.37
Corticosteroid use before the index hospitalization	18 (7)	72 (9)	0.42
Lifetime history of systemic antibiotic use	79 (32)	239 (31)	0.74
**Clinical presentation**			
Body weight at presentation (kg), median (IQR)	6.1 (4.6-8.0)	6.1 (4.7-7.7)	0.93
Respiratory rate at presentation (per minute), median (IQR)	48 (40-60)	48 (40-60)	0.95
Oxygen saturation at presentation			0.13
<90%	18 (7)	73 (9)	
90-93%	29 (12)	126 (16)	
≥94%	190 (78)	559 (72)	
**Respiratory virus**			
Any RSV	222 (91)	599 (78)	<0.001
Any rhinovirus	51 (21)	163 (21)	0.99
Other coinfection pathogens*	47 (19)	190 (25)	0.10
**Clinical outcomes**			
Positive pressure ventilation during hospitalization‡	18 (7)	37 (5)	0.16
Intensive care use during hospitalization§	42 (17)	121 (16)	0.64
Hospital length of stay (day), median (IQR)	2 (1-3)	2 (1-3)	0.22

Abbreviations: IQR, interquartile range; RSV, respiratory syncytial virus.

Data are n (%) of infants unless otherwise indicated. Percentages may not equal 100 because of rounding and missingness. Chi-square tests were used to compare proportions for categorical variables (such as sex), and Mann–Whitney *U* tests to compare median for continuous variables (such as age).

[*] Defined as an infant having a cough that wakes him or her at night or causes emesis, or when the child has wheezing or shortness of breath without cough.
[†] Adenovirus, bocavirus, *Bordetella pertussis*, enterovirus, human coronavirus NL63, OC43, 229E, or HKU1, human metapneumovirus, influenza A or B virus, *Mycoplasma pneumoniae*, and parainfluenza virus 1-3.
[‡] Defined as use of invasive and/or non-invasive mechanical ventilation (e.g., continuous positive airway pressure ventilation).
[§] Defined as use of positive pressure ventilation and/or intensive care unit admission.

Supplemental Table 3. Pathway enrichment analysis of each module of weighted gene co-expression network analysis in host response

Host response module	Number of assigned genes	GO ID	Category	Pathway*	FDR†	Correlation coefficient ‡ (P-value)	
						PPV	Intensive care treatment
**T cell regulation**	96	GO:0002250	BP	Adaptive immune response	<0.001	-0.24 (<0.001)	-0.23 (<0.001)
		GO:0042110	BP	T cell activation	<0.001		
		GO:0051249	BP	Regulation of lymphocyte activation	<0.001		
		GO:0050851	BP	Antigen receptor-mediated signaling pathway	<0.001		
		GO:0050863	BP	Regulation of T cell activation	<0.001		
		GO:0030098	BP	Lymphocyte differentiation	<0.001		
		GO:0030217	BP	T cell differentiation	<0.001		
		GO:0050852	BP	T cell receptor signaling pathway	<0.001		
		GO:0050870	BP	Positive regulation of T cell activation	<0.001		
		GO:0045580	BP	Regulation of T cell differentiation	<0.001		
		GO:0002429	BP	Immune response-activating cell surface receptor signaling pathway	<0.001		
		GO:0002757	BP	Immune response-activating signal transduction	<0.001		
		GO:0045619	BP	Regulation of lymphocyte differentiation	<0.001		
		GO:0002768	BP	Immune response-regulating cell surface receptor signaling pathway	<0.001		
		GO:0002764	BP	Immune response-regulating signaling pathway	<0.001		
		GO:0002253	BP	Activation of immune response	<0.001		
		GO:0051251	BP	Positive regulation of lymphocyte activation	<0.001		

		GO:1903039	BP	Positive regulation of leukocyte cell-cell adhesion	<0.001		
		GO:0050671	BP	Positive regulation of lymphocyte proliferation	<0.001		
		GO:0046651	BP	Lymphocyte proliferation	<0.001		
**Neutrophil/IL-1**	156	GO:0071347	BP	Cellular response to interleukin-1	<0.001	0.23 (<0.001)	0.26 (<0.001)
		GO:0070555	BP	Response to interleukin-1	<0.001		
		GO:0030593	BP	Neutrophil chemotaxis	<0.001		
		GO:0097530	BP	Granulocyte migration	<0.001		
		GO:1990266	BP	Neutrophil migration	<0.001		
		GO:0002819	BP	Regulation of adaptive immune response	<0.001		
		GO:0071621	BP	Granulocyte chemotaxis	<0.001		
		GO:0046456	BP	Icosanoid biosynthetic process	<0.001		
		GO:0060759	BP	Regulation of response to cytokine stimulus	<0.001		
		GO:0030728	BP	Ovulation	<0.001		
		GO:0006636	BP	Unsaturated fatty acid biosynthetic process	<0.001		
		GO:0033089	BP	Positive regulation of T cell differentiation in thymus	<0.001		
		GO:0032006	BP	Regulation of tor signaling	<0.001		
		GO:0006089	BP	Lactate metabolic process	<0.001		
		GO:0071634	BP	Regulation of transforming growth factor beta production	<0.001		
		GO:0071604	BP	Transforming growth factor beta production	<0.001		
		GO:0070821	CC	Tertiary granule membrane	<0.001		
		GO:0070820	CC	Tertiary granule	<0.001		
		GO:0004896	MF	Cytokine receptor activity	<0.001		
		GO:0140375	MF	Immune receptor activity	<0.001		

**GPCR**	60	GO:0035256	MF	G protein-coupled glutamate receptor binding	0.002	-0.21 (0.001)	-0.20 (<0.001)
		GO:0071889	MF	14-3-3 protein binding	0.007		
		GO:0004864	MF	Protein phosphatase inhibitor activity	0.008		
**Type I IFN**	73	GO:0032606	BP	Type I interferon production	<0.001	-0.21 (0.001)	-0.21 (0.001)
		GO:0032479	BP	Regulation of type I interferon production	<0.001		
		GO:0060337	BP	Type I interferon signaling pathway	<0.001		
		GO:0071357	BP	Cellular response to type I interferon	<0.001		
		GO:0034340	BP	Response to type I interferon	<0.001		
		GO:0051607	BP	Defense response to virus	<0.001		
		GO:0009615	BP	Response to virus	<0.001		
		GO:0034341	BP	Response to interferon-gamma	<0.001		
		GO:0060333	BP	Interferon-gamma-mediated signaling pathway	<0.001		
		GO:0071346	BP	Cellular response to interferon-gamma	<0.001		
		GO:1903900	BP	Regulation of viral life cycle	<0.001		
		GO:1903901	BP	Negative regulation of viral life cycle	<0.001		
		GO:0048525	BP	Negative regulation of viral process	<0.001		
		GO:0050792	BP	Regulation of viral process	<0.001		
		GO:0035455	BP	Response to interferon-alpha	<0.001		
		GO:0045069	BP	Regulation of viral genome replication	<0.001		
		GO:0045071	BP	Negative regulation of viral genome replication	<0.001		
		GO:0019079	BP	Viral genome replication	<0.001		
		GO:0046596	BP	Regulation of viral entry into host cell	<0.001		

		GO:1902187	BP	Negative regulation of viral release from host cell	<0.001		
**HR-1**	194	GO:0030863	CC	Cortical cytoskeleton	0.001	-0.21 (0.001)	-0.17 (0.006)
		GO:0000313	CC	Organellar ribosome	0.001		
		GO:0005761	CC	Mitochondrial ribosome	0.001		
		GO:0005884	CC	Actin filament	0.004		
		GO:0030864	CC	Cortical actin cytoskeleton	0.004		
		GO:0000315	CC	Organellar large ribosomal subunit	0.006		
		GO:0005762	CC	Mitochondrial large ribosomal subunit	0.006		
		GO:0097197	CC	Tetraspanin-enriched microdomain	0.007		
		GO:0031941	CC	Filamentous actin	0.007		
		GO:0001725	CC	Stress fiber	0.007		
		GO:0097517	CC	Contractile actin filament bundle	0.007		
		GO:0031094	CC	Platelet dense tubular network	0.007		
		GO:0032432	CC	Actin filament bundle	0.009		
		GO:0042641	CC	Actomyosin	0.009		
		GO:0016327	CC	Apicolateral plasma membrane	0.02		
**ATP synthesis**	57	GO:0005753	CC	Mitochondrial proton-transporting ATP synthase complex	0.01	0.20 (0.002)	0.17 (0.008)
		GO:0045259	CC	Proton-transporting ATP synthase complex	0.01		
		GO:0046933	MF	Proton-transporting ATP synthase activity, rotational mechanism	0.01		
		GO:0015252	MF	Proton channel activity	0.01		
**HR-2**	135	GO:0043001	BP	Golgi to plasma membrane protein transport	0.002	-0.19 (0.003)	-0.18 (0.004)
		GO:0002523	BP	Leukocyte migration involved in inflammatory response	0.002		
		GO:0051693	BP	Actin filament capping	0.002		

		GO:0030835	BP	Negative regulation of actin filament depolymerization	0.002		
		GO:1901879	BP	Regulation of protein depolymerization	0.002		
		GO:0030834	BP	Regulation of actin filament depolymerization	0.003		
		GO:0030042	BP	Actin filament depolymerization	0.003		
		GO:0006893	BP	Golgi to plasma membrane transport	0.003		
		GO:0061951	BP	Establishment of protein localization to plasma membrane	0.003		
		GO:0030837	BP	Negative regulation of actin filament polymerization	0.003		
		GO:0098840	BP	Protein transport along microtubule	0.003		
		GO:0099118	BP	Microtubule-based protein transport	0.003		
		GO:0010644	BP	Cell communication by electrical coupling	0.003		
		GO:1901880	BP	Negative regulation of protein depolymerization	0.003		
		GO:0016529	CC	Sarcoplasmic reticulum	0.003		
		GO:0032272	BP	Negative regulation of protein polymerization	0.004		
		GO:1900747	BP	Negative regulation of vascular endothelial growth factor signaling pathway	0.004		
		GO:0043242	BP	Negative regulation of protein-containing complex disassembly	0.004		
		GO:0016528	CC	Sarcoplasm	0.004		
**Transcription-translation**	163	GO:0000466	BP	Maturation of 5.8s rRNA from tricistronic rRNA transcript (ssu-rRNA, 5.8s rRNA, lsu-rRNA)	0.001	-0.18 (0.005)	-0.18 (0.005)
		GO:1903313	BP	Positive regulation of mRNA metabolic process	0.001		

		GO:0030684	CC	Preribosome	0.001		
		GO:0030686	CC	90s preribosome	0.001		
		GO:0005684	CC	U2-type spliceosomal complex	0.001		
		GO:0070273	MF	Phosphatidylinositol-4-phosphate binding	0.001		
		GO:0042054	MF	Histone methyltransferase activity	0.001		
		GO:0000460	BP	Maturation of 5.8s rRNA	0.002		
		GO:0010314	MF	Phosphatidylinositol-5-phosphate binding	0.004		
		GO:0018027	BP	Peptidyl-lysine dimethylation	0.005		
		GO:0048026	BP	Positive regulation of mRNA splicing, via spliceosome	0.005		
		GO:0008276	MF	Protein methyltransferase activity	0.005		
		GO:0043325	MF	Phosphatidylinositol-3,4-bisphosphate binding	0.008		
		GO:0048188	CC	Set1c/compass complex	0.01		
		GO:0071006	CC	U2-type catalytic step 1 spliceosome	0.01		
		GO:0010494	CC	Cytoplasmic stress granule	0.01		
		GO:0030291	MF	Protein serine/threonine kinase inhibitor activity	0.01		
		GO:0030515	MF	snoRNA binding	0.01		
		GO:0034511	MF	U3 snoRNA binding	0.01		
		GO:0042162	MF	Telomeric DNA binding	0.01		
**Granulocyte**	128	GO:0070820	CC	Tertiary granule	<0.001	0.13 (0.04)	0.13 (0.04)
		GO:0042581	CC	Specific granule	<0.001		
		GO:0070821	CC	Tertiary granule membrane	<0.001		
		GO:0035579	CC	Specific granule membrane	<0.001		
		GO:1904724	CC	Tertiary granule lumen	<0.001		
		GO:0043312	BP	Neutrophil degranulation	<0.001		
		GO:0002283	BP	Neutrophil activation involved in immune response	<0.001		

GO:0002446	BP	Neutrophil mediated immunity	<0.001
GO:0042119	BP	Neutrophil activation	<0.001
GO:0032496	BP	Response to lipopolysaccharide	<0.001
GO:0071222	BP	Cellular response to lipopolysaccharide	<0.001
GO:0071219	BP	Cellular response to molecule of bacterial origin	<0.001
GO:0071216	BP	Cellular response to biotic stimulus	<0.001
GO:0002573	BP	Myeloid leukocyte differentiation	<0.001
GO:0002761	BP	Regulation of myeloid leukocyte differentiation	<0.001
GO:1904705	BP	Regulation of vascular associated smooth muscle cell proliferation	<0.001
GO:1990874	BP	Vascular associated smooth muscle cell proliferation	<0.001
GO:0071621	BP	Granulocyte chemotaxis	<0.001
GO:0048661	BP	Positive regulation of smooth muscle cell proliferation	<0.001
GO:1903977	BP	Positive regulation of glial cell migration	<0.001

Abbreviations: ATP, adenosine triphosphate; BP, biological process; CC, cellular component; FDR, false discovery rate; HR, host response; IFN, interferon; IL, interleukin; MF, molecular function; PPV, positive pressure ventilation

[*] The top 20 pathways with the highest FDR or pathways with P-values of <0.05 are shown.
[†] FDR is calculated based on the p-value of the pathway enrichment analysis in each module.
[‡] Spearman's coefficients between the eigenvalue (the first principal component) of corresponding module and PPV or intensive care use were estimated.

Supplemental Table 4. Assigned microbial species of each module of weighted gene co-expression network analysis in nasopharyngeal airway microbial composition

**Microbial composition module**	**Number of assigned species**	**Microbial species***	**Correlation coefficient[†] (P- value)**	
			**PPV**	**Intensive care treatment**
S. pneumonia/S. aureus	4	*Streptococcus pneumoniae, Staphylococcus aureus, Escherichia coli, Lactobacillus vaginalis*	0.16 (0.01)	0.12 (0.05)
**MC-1**	151	*Abiotrophia defectiva, Achromobacter xylosoxidans, Acidovorax ebreus, Acidovorax temperans, Acinetobacter baumannii, Acinetobacter johnsonii, Acinetobacter junii, Acinetobacter lwoffii, Acinetobacter radioresistens, Actinomyces dentalis, Actinomyces graevenitzii, Actinomyces johnsonii, Actinomyces massiliensis, Actinomyces naeslundii, Actinomyces viscosus, Aerococcus viridan, Afipia broomeae, Agrobacterium tumefaciens, Alloiococcus otitis, Anaerococcus prevotii, Anaerococcus tetradius, Anoxybacillus flavithermus, Arsenicicoccus bolidensis, Arthrospira platensis, Atopobium parvulum, Atopobium vaginae, Bacillus anthracis, Bacillus clausii, Bacillus subtilis, Bifidobacterium animalis, Bifidobacterium longum Bordetella pertussis, Bradyrhizobium elkanii, Brevundimonas diminuta, Brochothrix thermosphacta, Burkholderia cepacia, Capnocytophaga leadbetteri, Cardiobacterium hominis, Comamonas testosteroni, Corynebacterium accolens, Corynebacterium afermentans, Corynebacterium amycolatum, Corynebacterium appendicis, Corynebacterium aurimucosum, Corynebacterium coyleae, Corynebacterium durum, Corynebacterium jeikeium, Corynebacterium kroppenstedtii, Corynebacterium matruchotii, Corynebacterium otitidis, Corynebacterium pilbarense, Corynebacterium simulans, Corynebacterium singulare, Corynebacterium striatum, Corynebacterium tuberculostearicum, Corynebacterium urealyticum, Cronobacter sakazakii, Cupriavidus gilardii, Cutibacterium acnes, Cutibacterium avidum, Cutibacterium granulosum, Delftia acidovorans, Dialister micraerophilus, Dietzia cinnamea, Dolosigranulum pigrum, Enhydrobacter aerosaccus, Enterobacter cancerogenus, Enterobacter hormaechei, Enterococcus durans, Enterococcus faecalis, Enterococcus italicus, Finegoldia magna, Gardnerella vaginalis, Granulicatella adiacens, Haematobacter missouriensis, Jonquetella anthropi, Klebsiella pneumoniae, Kluyvera ascorbata, Kocuria palustris, Kocuria rhizophila, Kytococcus sedentarius,*	0.11 (0.10)	0.022 (0.70)

		Lactobacillus acidophilus, Lactobacillus brevis, Lactobacillus casei, Lactobacillus crispatus, Lactobacillus fermentum, Lactobacillus gasseri, Lactobacillus iners, Lactobacillus plantarum, Lactobacillus rhamnosus, Lactobacillus salivarius, Lactococcus lactis, Lautropia mirabilis, Lawsonella clevelandensis, Listeria monocytogenes, Lysinibacillus fusiformis, Megasphaera micronuciformis, Mesorhizobium loti, Microbacterium flavescens, Microbacterium ginsengisoli, Micrococcus luteus, Mitsuokella multacida, Mobiluncus mulieris, Moraxella osloensis, Mycobacterium tuberculosis, Mycolicibacterium neoaurum, Neisseria sicca, Neisseria subflava, Ochrobactrum anthropi, Paenibacillus glucanolyticus, Paenibacillus phoenicis, Paracoccus yeei, Peptidiphaga gingivicola, Peptoniphilus harei, Peptostreptococcus anaerobius, Prevotella buccalis, Proteus mirabilis, Pseudomonas aeruginosa, Pseudomonas fluorescens, Pseudomonas oleovoran, Pseudomonas stutzeri, Pseudopropionibacterium propionicum, Pseudoramibacter alactolyticus, Ralstonia pickettii, Roseomonas gilardii, Roseomonas mucosa, Rothia aeria, Rothia dentocariosa, Rothia mucilaginosa, Sanguibacter keddieii, Schaalia odontolytica, Serratia marcescens, Staphylococcus capitisStaphylococcus caprae, Staphylococcus cohnii, Staphylococcus epidermidis, Staphylococcus hominis, Staphylococcus pettenkoferi, Staphylococcus warneri, Stenotrophomonas maltophilia, Stenotrophomonas nitritireducens, Streptococcus australis, Streptococcus gordonii, Streptococcus salivarius, Streptococcus sanguinis, Streptococcus thermophilus, Treponema vincentii, Variovorax paradoxus, Veillonella parvula, Yersinia pestis		
Moraxella	3	*Moraxella catarrhalis, Moraxella lincolnii, Moraxella nonliquefaciens*	-0.09 (0.10)	-0.07 (0.30)
Streptococcus	13	*Streptococcus agalactiae, Streptococcus anginosus, Streptococcus constellatus, Streptococcus downei, Streptococcus intermedius, Streptococcus mutans, Streptococcus oralis, Streptococcus peroris, Streptococcus pyogenes, Streptococcus sinensis, Streptococcus sobrinus, Streptococcus vestibularis, Enterococcus saccharolyticus*	0.08 (0.20)	0.05 (0.40)
Haemophilus	5	*Haemophilus aegyptius, Haemophilus ducreyi, Haemophilus influenzae, Haemophilus paraphrohaemolyticus, Haemophilus pittmaniae*	0.05 (0.40)	0.0005 (0.99)

**MC-2**	105	*Aggregatibacter aphrophilus, Alloprevotella rava, Alloprevotella tannerae, Alloscardovia omnicolens, Anaerococcus lactolyticus, Bacteroides pyogenes, Bulleidia extructa, Campylobacter concisus, Campylobacter rectus, Campylobacter showae, Capnocytophaga gingivalis, Capnocytophaga granulosa, Capnocytophaga haemolytica, Capnocytophaga ochracea, Capnocytophaga sputigena, Catonella morbi, Eggerthia catenaformis, Eikenella corrodens, Fusobacterium gonidiaformans, Fusobacterium hwasookii, Fusobacterium nucleatum, Fusobacterium periodonticum, Gemella haemolysans, Gemella morbillorum, Gemella sanguinis, Granulicatella elegans, Haemophilus haemolyticus, Haemophilus parahaemolyticus, Haemophilus parainfluenzae, Haemophilus sputorum, Johnsonella ignava, Kingella denitrificans, Kingella kingae, Kingella oralis, Lachnoanaerobaculum orale, Lachnoanaerobaculum saburreum, Lachnoanaerobaculum umeaense, Leptotrichia buccalis, Leptotrichia goodfellowii, Leptotrichia hofstadii, Leptotrichia shahii, Leptotrichia wadei, Mogibacterium pumilum, Neisseria bacilliformis, Neisseria cinerea, Neisseria elongata, Neisseria flavescens, Neisseria gonorrhoeae, Neisseria lactamica, Neisseria macacae, Neisseria meningitidis, Neisseria mucosa, Neisseria oralis, Neisseria polysaccharea, Neisseria weaveri, Oribacterium asaccharolyticum, Oribacterium parvum, Parvimonas micra, Peptoniphilus lacrimalis, Peptostreptococcus stomatis, Porphyromonas asaccharolytica, Porphyromonas catoniae, Porphyromonas endodontalis, Porphyromonas gingivalis, Porphyromonas pasteri, Porphyromonas uenonis, Prevotella baroniae, Prevotella bivia, Prevotella buccae, Prevotella denticola, Prevotella enoeca, Prevotella fusca, Prevotella histicola, Prevotella intermedia, Prevotella loescheii, Prevotella maculosa, Prevotella marshii, Prevotella melaninogenica, Prevotella micans, Prevotella multiformis, Prevotella nanceiensis, Prevotella nigrescens, Prevotella oralis, Prevotella oris, Prevotella oulorum, Prevotella pallens, Prevotella pleuritidis, Prevotella saccharolytica, Prevotella salivae, Prevotella scopos, Prevotella shahii, Prevotella veroralis, Schaalia meyeri, Selenomonas infelix, Selenomonas sputigena, Shuttleworthia satelles, Simonsiella muelleri, Solobacterium moorei, Stomatobaculum longum, Streptococcus mitis, Tannerella forsythia, Veillonella atypica, Veillonella denticariosi, Veillonella dispar, Veillonella rogosae*	-0.05 (0.50)	-0.04 (0.60)
**MC-3**	3	*Caldilinea aerophila, Desulfomicrobium orale, Micavibrio aeruginosavorus*	0.02 (0.70)	-0.03 (0.70)

3 Abbreviations: MC, microbial composition; PPV, positive pressure ventilation

[*] All assigned microbial species are shown.

[†] Spearman's correlation coefficients between the eigenvalue (the first principal component) of corresponding module and PPV or intensive care use were estimated.

Supplemental Table 5. Pathway enrichment analysis of each module of weighted gene co-expression network analysis in nasopharyngeal airway microbial function

Microbial function module	Number of assigned genes	GO ID	Category	Pathway*	FDR†	Correlation coefficient ‡ (P- value)	
						PPV	Intensive treatment care
**Plasma membrane**	367	GO:0031226	CC	Intrinsic component of plasma membrane	0.002	-0.36 (<0.001)	-0.24 (<0.001)
		GO:0005887	CC	Integral component of plasma membrane	0.002		
**mRNA metabolism**	229	GO:0006402	BP	mRNA catabolic process	<0.001	-0.23 (<0.001)	-0.11 (0.07)
		GO:0016071	BP	mRNA metabolic process	<0.001		
		GO:0006401	BP	RNA catabolic process	0.001		
		GO:0010605	BP	Negative regulation of macromolecule metabolic process	0.002		
		GO:0009892	BP	Negative regulation of metabolic process	0.002		
		GO:0016070	BP	RNA metabolic process	0.002		
		GO:0010467	BP	Gene expression	0.002		
**BCAA metabolism**	10	GO:0009081	BP	Branched-chain amino acid metabolic process	0.03	-0.19 (0.004)	-0.07 (0.249)
		GO:0009082	BP	Branched-chain amino acid biosynthetic process	0.03		
		GO:0006549	BP	Isoleucine metabolic process	0.03		
		GO:0009097	BP	Isoleucine biosynthetic process	0.03		
		GO:0045271	CC	Respiratory chain complex I	0.03		
		GO:0045272	CC	Plasma membrane respiratory chain complex I	0.03		
		GO:0030964	CC	NADH dehydrogenase complex	0.03		
		GO:0050136	MF	NADH dehydrogenase (quinone) activity	0.03		

		GO:0008137	MF	NADH dehydrogenase (ubiquinone) activity	0.03		
		GO:0003954	MF	NADH dehydrogenase activity	0.03		
		GO:0048038	MF	Quinone binding	0.03		
		GO:0016655	MF	Oxidoreductase activity, acting on NAD(P)H, quinone or similar compound as acceptor	0.03		
**Oxidative stress response**	5	GO:0034599	BP	Cellular response to oxidative stress	0.03	-0.16 (0.01)	-0.06 (0.28)
		GO:0098869	BP	Cellular oxidant detoxification	0.03		
		GO:1901701	BP	Cellular response to oxygen-containing compound	0.03		
		GO:0097237	BP	Cellular response to toxic substance	0.03		
		GO:1990748	BP	Cellular detoxification	0.03		
		GO:0045454	BP	Cell redox homeostasis	0.03		
		GO:1901698	BP	Response to nitrogen compound	0.03		
		GO:0043086	BP	Negative regulation of catalytic activity	0.03		
		GO:0050790	BP	Regulation of catalytic activity	0.03		
		GO:0000028	BP	Ribosomal small subunit assembly	0.03		
		GO:0044092	BP	Negative regulation of molecular function	0.03		
		GO:0072657	BP	Protein localization to membrane	0.03		
		GO:0006935	BP	Chemotaxis	0.03		
		GO:0042330	BP	Taxis	0.03		
		GO:0019220	BP	Regulation of phosphate metabolic process	0.03		
		GO:0051174	BP	Regulation of phosphorus metabolic process	0.03		
		GO:0010243	BP	Response to organonitrogen compound	0.03		
		GO:0032879	BP	Regulation of localization	0.03		
		GO:0016209	MF	Antioxidant activity	0.03		
		GO:0004857	MF	Enzyme inhibitor activity	0.03		
		GO:0004601	MF	Peroxidase activity	0.03		

		GO:0016684	MF	Oxidoreductase activity, acting on peroxide as acceptor	0.03		
		GO:0038023	MF	Signaling receptor activity	0.03		
**NADH**	7	GO:0016655	MF	Oxidoreductase activity, acting on NAD(P)H, quinone or similar compound as acceptor	0.02	-0.13 (0.04)	-0.09 (0.15)
		GO:0008137	MF	NADH dehydrogenase (ubiquinone) activity	0.02		
		GO:0050136	MF	NADH dehydrogenase (quinone) activity	0.02		
		GO:0003954	MF	NADH dehydrogenase activity	0.02		
		GO:0008649	MF	rRNA methyltransferase activity	0.02		
		GO:0140102	MF	Catalytic activity, acting on a rRNA	0.02		
		GO:0048038	MF	Quinone binding	0.02		
		GO:0008171	MF	O-methyltransferase activity	0.02		
		GO:0045271	CC	Respiratory chain complex I	0.02		
		GO:0045272	CC	Plasma membrane respiratory chain complex I	0.02		
		GO:0030964	CC	NADH dehydrogenase complex	0.02		
		GO:0031167	BP	rRNA methylation	0.02		
**Protein secretion**	7	GO:0009306	BP	Protein secretion	0.02	0.001 (0.99)	0.13 (0.049)
		GO:0002790	BP	Peptide secretion	0.02		
		GO:0032940	BP	Secretion by cell	0.02		
		GO:0046903	BP	Secretion	0.02		
		GO:0035592	BP	Establishment of protein localization to extracellular region	0.02		
		GO:0071692	BP	Protein localization to extracellular region	0.02		
		GO:0071806	BP	Protein transmembrane transport	0.02		
**MF-1**	17	No assigned				-0.16 (0.01)	-0.05 (0.40)

**MF-2**	12	GO:0015179	MF	L-amino acid transmembrane transporter activity	0.03	-0.14 (0.03)	-0.09 (0.18)

Abbreviations: BP, biological process; CC, cellular component; FDR, false discovery rate; HR, host response; IFN, interferon; IL, interleukin; NADH, nicotinamide adenine dinucleotide; MF (in GO), molecular function; MF (in modules), microbial function; PPV, positive pressure ventilation

[*] The top 20 pathways with the highest FDR or pathways with P-values of <0.05 are shown.
[†] FDR is calculated based on the p-value of the pathway enrichment analysis in each module.
[‡] Spearman's coefficients between the eigenvalue (the first principal component) of corresponding module and PPV or intensive care use were estimated.

Supplemental Table 6. Primers for respiratory pathogen testing

Pathogen	Target gene	Primer name	Primer/Probe Sequence
RSV A	Nucleoprotein	RSVA Fwd.	AGA TCA ACT TCT GTC ATC CAG CAA
		RSVA Rev.	TTC TGC ACA TCA TAA TTA GGA GTA TCA AT
		RSVA Probe	CAC CAT CCA ACG GAG CAC AGG AGA T
RSV B	Nucleoprotein	RSVB Fwd.	AAG ATG CAA ATC ATA AAT TCA CAG GA
		RSVB Rev.	TGA TAT CCA GCA TCT TTA AGT ATC TTT ATA GTG
		RSVB Probe	AGG TAT GTT ATA TGC TAT GTC CAG GTT AGG AAG GGA A
RSV A*	Nucleoprotein	LTRSVA Fwd.	GATACACTCAACAAAGATCAACTTCTGTCA
		LTRSVA Rev.	AGGAGTGTCAATGCTGTCTCCTGTG
		LT RSVA Probe	TCCAGCAAATACACCATCCAACGGAG
RSV B*	Nucleoprotein	LTRSVB Fwd.	CTGTGTATAGCTGCCCTTGTAATAACC
		LTRSVB Rev.	GACATTGTTTGCCCTCCTAATTACTGC
		LTRSVB Probe	AGCAGCAGGAGATAGATCAGGTCTTACA
Influenza A	Matrix	FluA Fwd.	GAC CRA TCC TGT CAC CTC TGA
		FluA Rev.	AGG GCA TTY TGG ACA AAK CGT CTA
		FluA Probe	TGC AGT CCT CGC TCA CGT GGC ACG
Influenza B	Nucleoprotein	FluB Fwd.	AAG ACC TRA GAG TTT TGT CTG CAY T
		FluB Rev.	ATC AGA GCT GCY CCC ATT
		FluB Probe	TGC AAG GGT TTC CAY GTT CCA GCA
Parainfluenza-1	Polymerase	PIV-1 Fwd.	ACA GAT GAA ATT TTC AAG TGC TAC TTT AGT
		PIV-1 Rev.	GCC TCT TTT AAT GCC ATA TTA TCA TTA GA
		PIV-1 Probe	ATG GTA ATA AAT CGA CTC GCT
Parainfluenza-2	Polymerase	PIV-2 Fwd.	TGC ATG TTT TAT AAC TAC TGA TCT TGC TAA
		PIV-2 Rev.	GTT CGA GCA AAA TGG ATT ATG GT
		PIV-2 Probe	ACT GTC TTC AAT GGA GAT AT
Parainfluenza-3	Nucleoprotein	PIV-3 Fwd.	TGT TGA GCC TAT TTG ATA CAT TTA ATG C
		PIV-3 Rev.	ATG ATA GCT CCA CCA GCT GAT TTT
		PIV-3 Probe	CGT AGG CAA GAA AAC ATA A
Rhinovirus	5’UTR	HRV Fwd	CY+ AGC C+T GCG TGG C (+ is LNA base)
		HRV Rev.	GAA ACA CGG ACA CCC AAA GTA
		HRV Probe	TCC TCC GGC CCC TGA ATG YGG C
Human Metapneumovirus	Nucleoprotein	HMPV Fwd.	CAT ATA AGC ATG CTA TAT TAA AAG AGT CTC
		HMPV Rev.	CCT ATT TCT GCA TAT TTG TAA TCA G
		HMPV Probe	TGY AAT GAT GAG GGT GTC ACT GCG GTT G
Enterovirus	5’UTR	Ent Fwd.	GAT TGT CAC CAT AAG CAG C
		Ent Rev	CCC CTG AAT GCG GCT AAT C
		Ent Probe	CGG AAC CGA CTA CTT TGG GTG TCC GT
Human Corona virus 229E	Nucleoprotein	229E Fwd.	TCT GCC AAG AGT CTT GCT CG
		229E Rev.	AGC ATA GCA GCT GTT GAC GG
		229E Probe	TGG CCA CAA CAC CTG CAC TTC C
Human Corona virus OC43	Nucleoprotein	OC43 Fwd.	CAT CAG GAG GGA ATG TTG TAC C
		OC43 Rev.	TAC TGG TCT TTA GCA TGC GGT C
		OC43 Probe	CAG CAG TTG ACG CTG GTT GCC ATC

Human Corona virus HKU1	Replicase 1B	HKU1 Fwd.	CCT TGC GAA TGA ATG TGC T
		HKU1 Rev.	TTG CAT CAC CAC TGC TAG TAC CAC
		HKU1 Probe	TGT GTG GCG GTT GCT ATT ATG TTA AGC CTG
Human Corona virus NL63	Nucleoprotein	NL63 Fwd.	GAC CAA AGC ACT GAA TAA CAT TTT CC
		NL63 Rev.	ACC TAA TAA GCC TCT TTC TCA ACC C
		NL63 Probe	AAC ACG CTT CCA ACG AGG TTT CTT CAA CTG AG
Adenovirus	Hexon	Adeno Fwd.	CAG GAC GCC TCG GRG TAY CTS AG
		Adeno Rev.	GGA GCC ACV GTG GGR TT
		Adeno Probe	CCG GGT CTG GTG CAG TTT GCC C
Bocavirus	Non-structural protein 1	Boca Fwd.	TGC AGA CAA CGC YTA GTT GTT T
		Boca Rev.	CTG TCC CGC CCA AGA TAC A
		Boca Probe	CCA GGA TTG GGT GGA ACC TGC AAA
Mycoplasma pneumoniae	CARDS toxin	Myco Fwd.	TTT GGT AGC TGG TTA CGG GAA T
		Myco Rev.	GGT CGG CAC GAA TTT CAT ATA AG
		Myco Probe	TGT ACC AGA GCA CCC CAG AAG GGC T
Bordetella pertussis (Screening assay)	IS481	ISBordo Fwd.	GCG TGC AGA TTC GTC GTA C
		ISBordo Rev.	TGA TGG TGC CTA TTT TAC GG
		ISBordo Probe	ACC CTC GAT TCT TCC GT
Bordetella pertussis (Confirmatory assay)	Toxin	Bordo Fwd.	GCG TGC AGA TTC GTC GTA C
		Bordo Rev.	AGG GCA TTY TGG ACA AAK CGT CTA
		Bordo Probe	TGC AGT CCT CGC TCA CTG GGC ACG

Supplemental Figure 1. Study flow diagram

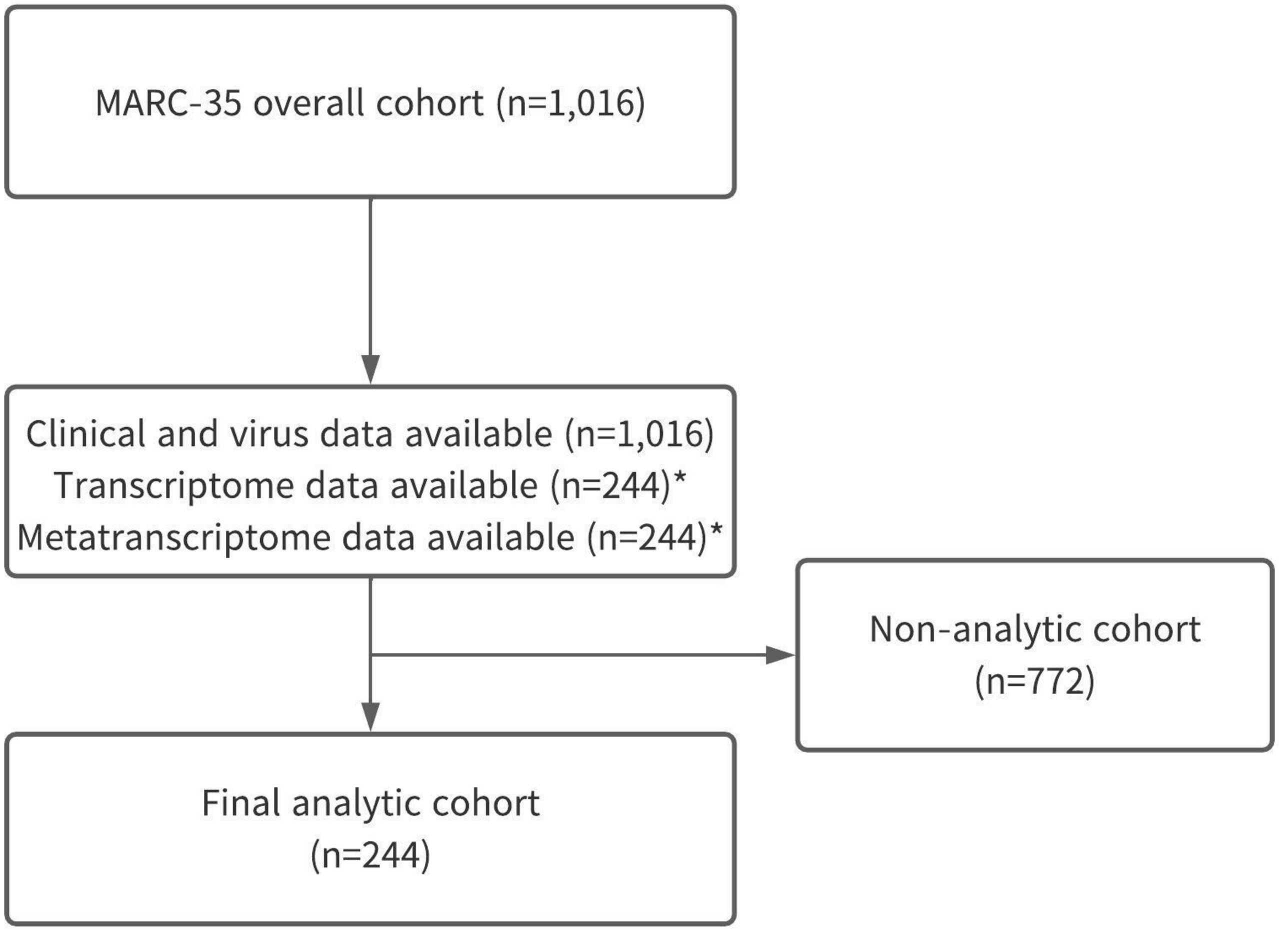

The differences in the analytic and non-analytic cohorts are summarized in **Supplemental Table 1**.
* The transcriptome and metatranscriptome data were obtained in 244 infants who were *randomly* selected from the overall cohort

Supplemental Figure 2. Gene set enrichment analysis of host transcriptome data with regard to the use of positive pressure ventilation in infants hospitalized for bronchiolitis

A)

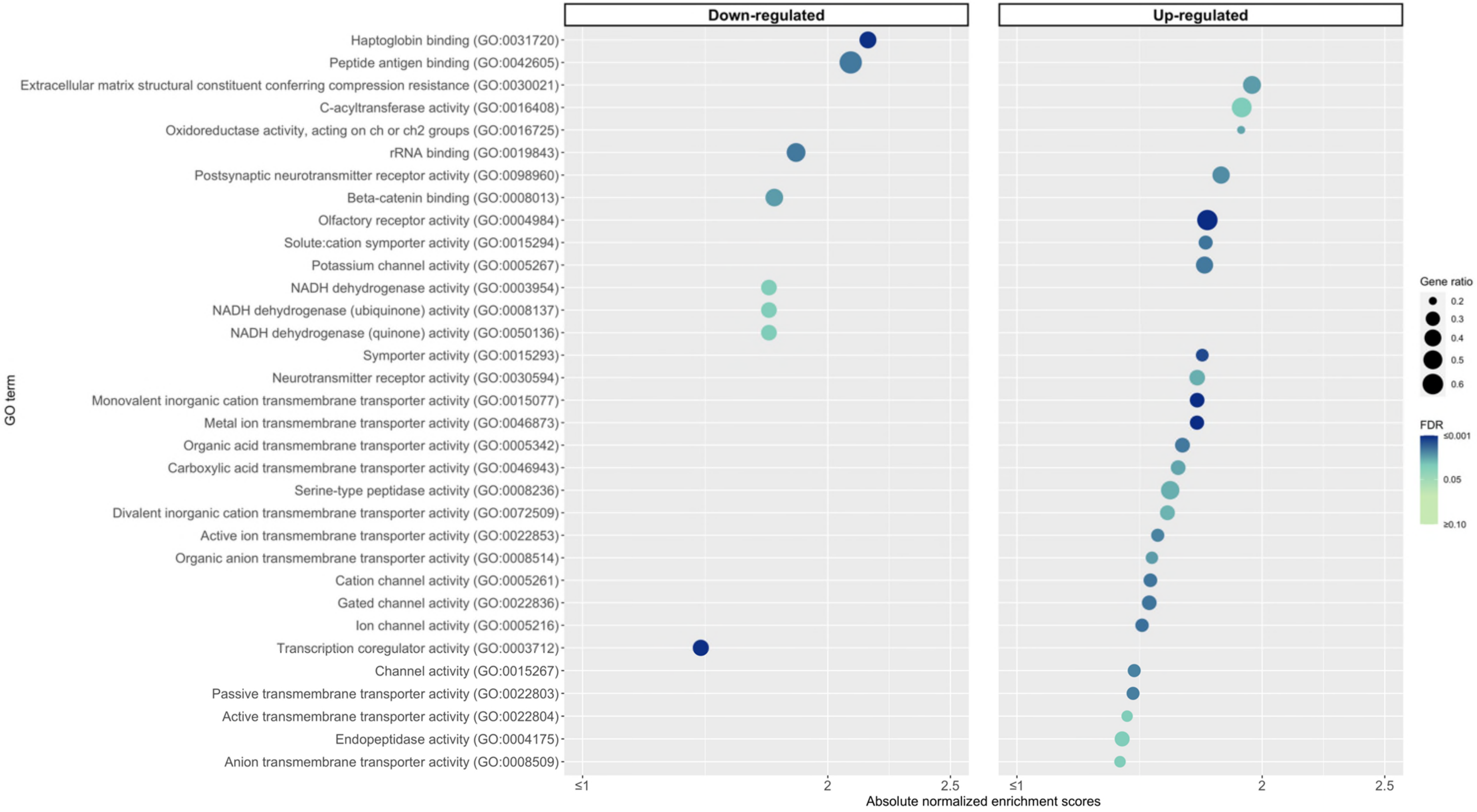

B)

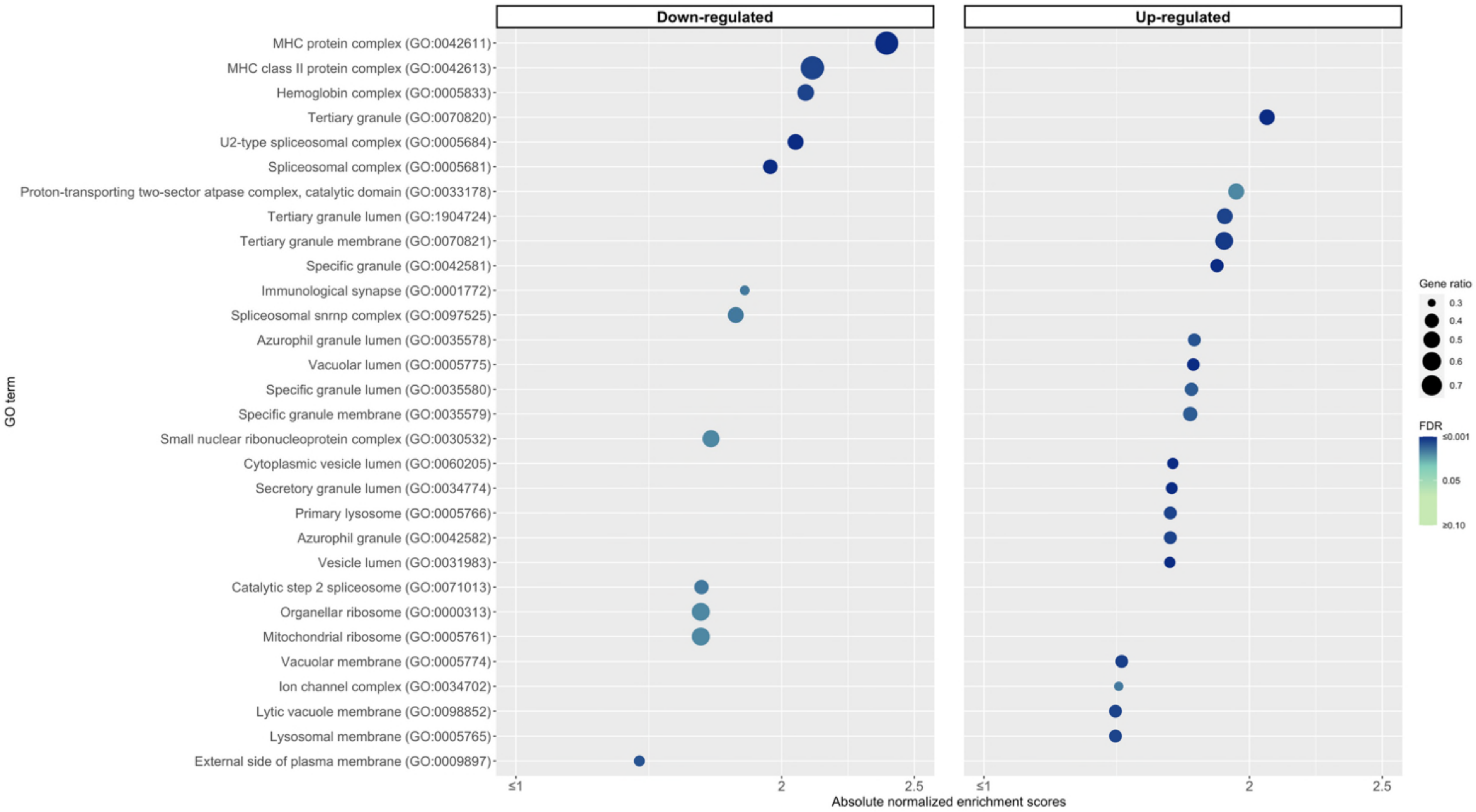
Down-regulated
Up-regulated
MHC protein complex (GO:0042611)
MHC class II protein complex (GO:0042613)
Hemoglobin complex (GO:0005833)
Tertiary granule (GO:0070820)
U2-type spliceosomal complex (GO:0005684)
Spliceosomal complex (GO:0005681)
Proton-transporting two-sector atpase complex, catalytic domain (GO:0033178)
Tertiary granule lumen (GO:1904724)
Tertiary granule membrane (GO:0070821)
Specific granule (GO:0042581)
Immunological synapse (GO:0001772)
Spliceosomal snrnp complex (GO:0097525)
Azurophil granule lumen (GO:0035578)
Vacuolar lumen (GO:0005775)
Specific granule lumen (GO:0035580)
Specific granule membrane (GO:0035579)
Small nuclear ribonucleoprotein complex (GO:0030532)
Cytoplasmic vesicle lumen (GO:0060205)
Secretory granule lumen (GO:0034774)
Primary lysosome (GO:0005766)
Azurophil granule (GO:0042582)
Vesicle lumen (GO:0031983)
Catalytic step 2 spliceosome (GO:0071013)
Organellar ribosome (GO:0000313)
Mitochondrial ribosome (GO:0005761)
Vacuolar membrane (GO:0005774)
Ion channel complex (GO:0034702)
Lytic vacuole membrane (GO:0098852)
Lysosomal membrane (GO:0005765)
External side of plasma membrane (GO:0009897)
GO term
≤1
2
2.5
≤1
2
2.5
Absolute normalized enrichment scores
Gene ratio
0.3
0.4
0.5
0.6
0.7
FDR
≤0.001
0.05
≥0.10

A) GO molecular function. **B)** GO cellular component.

We showed 30 host pathways with the most significant FDR in the gene set enrichment analysis (GSEA) with down-regulated pathways on the left side and up-regulated pathways on the right side. We also showed the absolute normalized enrichment score, FDR, and the gene ratio for the corresponding pathways.

Abbreviations: FDR, false discovery rate; GO, gene ontology; GSEA, gene set enrichment analysis; MHC, major histocompatibility complex; NADH, nicotinamide adenine dinucleotide; PPV, positive pressure ventilation

Supplemental Figure 3. Relationship of abundant fungal species with the risk of higher severity in infants hospitalized for bronchiolitis

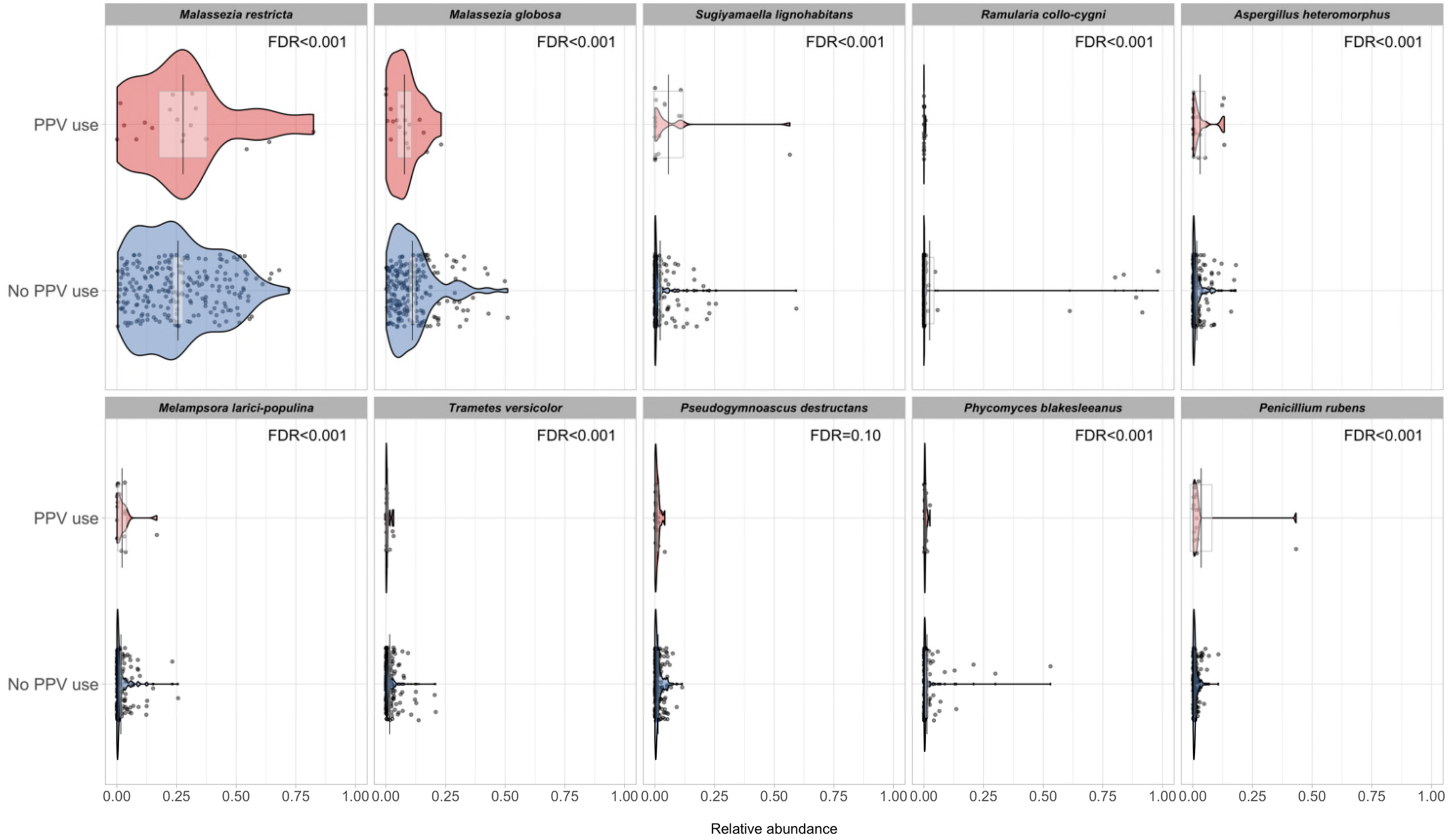

The pirate plots show the comparison of the distribution of annotated ten most abundant fungal species in the nasopharyngeal airway of infants hospitalized for bronchiolitis, according to the PPV use. Each point represents each infant. The grey bar and rectangle represent the mean and 95% confidence interval. In the violin plots, the width represents the probability that infants take on a specific relative abundance. The between-group differences in the abundance were tested by fitting Poisson regression models. n = 244 biologically independent samples.

Abbreviations: FDR, false discovery rate; PPV, positive pressure ventilation

Supplemental Figure 4. Gene set enrichment analysis of microbial function data with regard to the use of positive pressure ventilation in infants hospitalized for bronchiolitis
A)

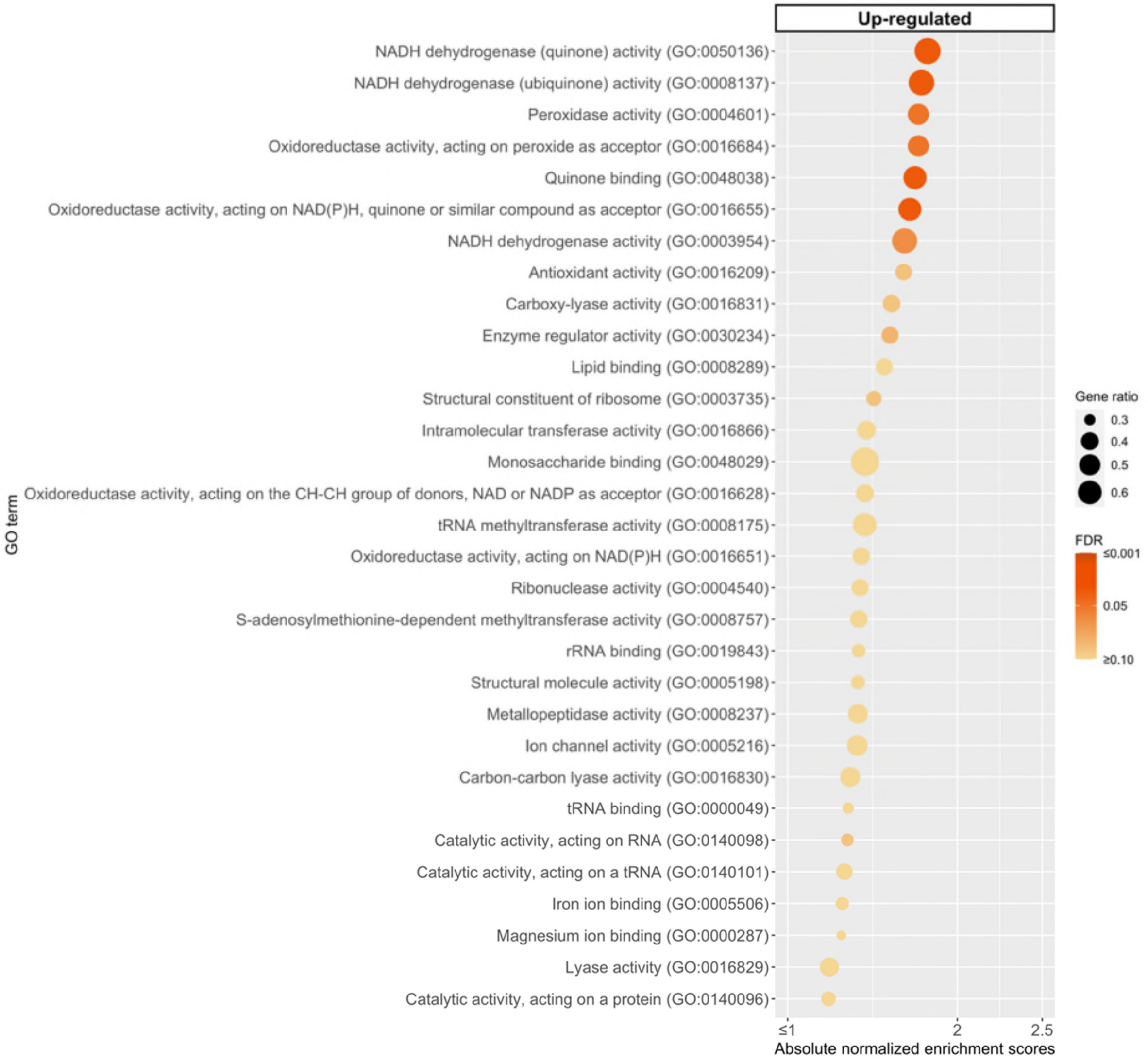

B)

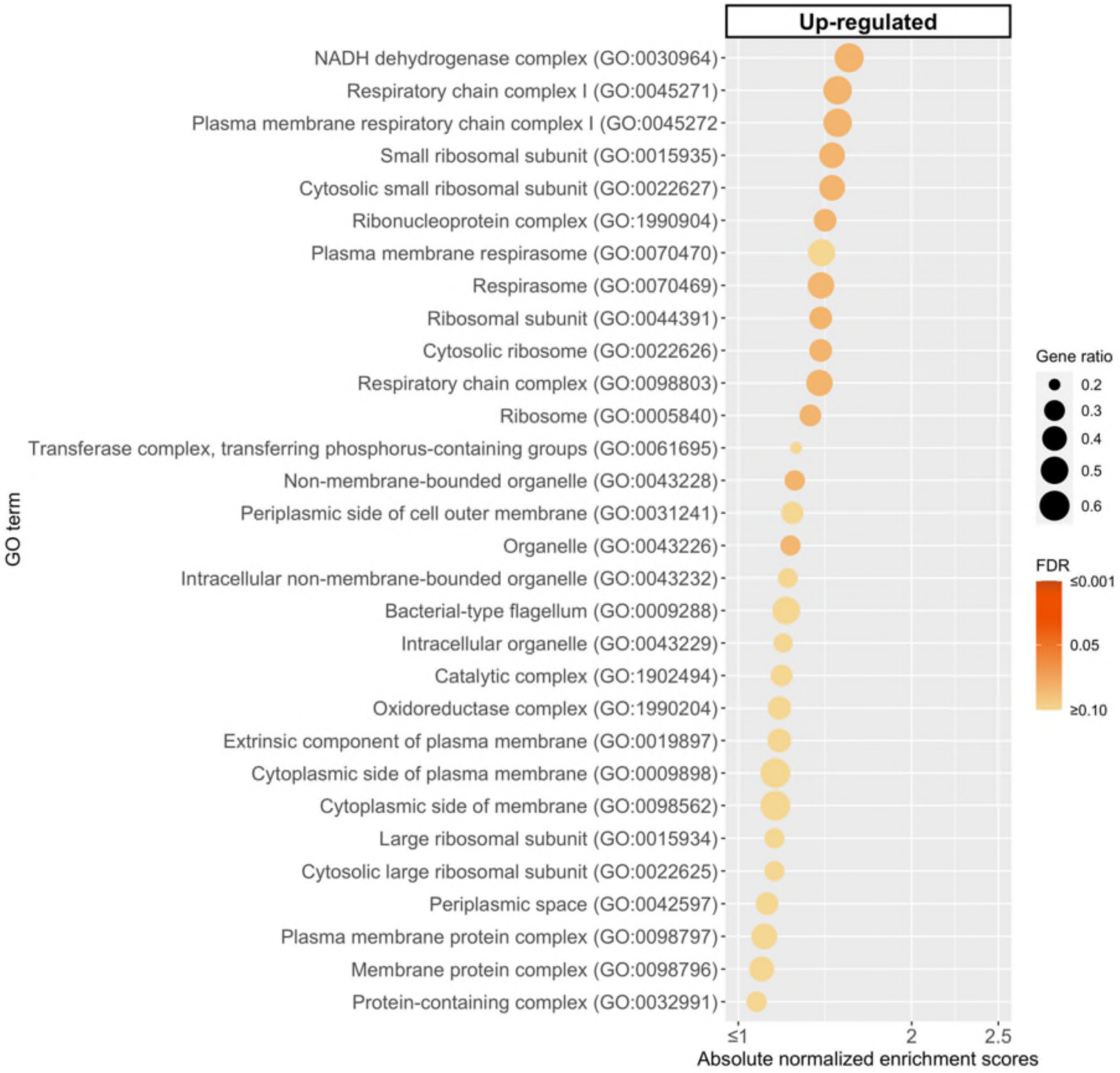

A) GO molecular function. **B)** GO cellular component. Gene set enrichment analysis (GSEA) of the metatranscriptome data. We showed 30 microbial functional pathways (GO biological process) with the most significant FDR in the gene set enrichment analysis (GSEA). Down-regulated pathways were not detected. We also showed the normalized enrichment score, FDR, and the gene ratio for the corresponding pathways.
Abbreviations: FDR, false discovery rate; GO, gene ontology; GSEA, gene set enrichment analysis NADH, nicotinamide adenine dinucleotide

Supplemental Figure 5. Sensitivity analysis: Integrated relationship of the dual-transcriptome modules with the risk of intensive care use in infants hospitalized for bronchiolitis

Module	Odds ratio (95% CI)
**T cell regulation module**	0.23 (0.08-0.62)
**Neutrophil/IL-1 module**	6.93 (2.48-21.6)
**GPCR module**	0.27 (0.09-0.69)
**Type I IFN module**	0.29 (0.09-0.72)
HR-1 module	0.44 (0.16-1.03)
S. pneumoniae/*S. aureus* module	2.06 (0.79-5.24)
MC-1 module	1.06 (0.41-2.73)
Moraxella module	0.64 (0.21-2.01)
Streptcoccus module	1.32 (0.47-3.84)
Haemophilus module	0.96 (0.32-2.87)
**Plasma membrane module**	0.33 (0.16-0.76)
mRNA metabolism module	0.51 (0.24-1.22)
BCAA metabolism module	0.71 (0.04-1.78)
Oxidative stress response module	0.64 (0.11-2.08)
**NADH module**	0.46 (0.02-0.71)

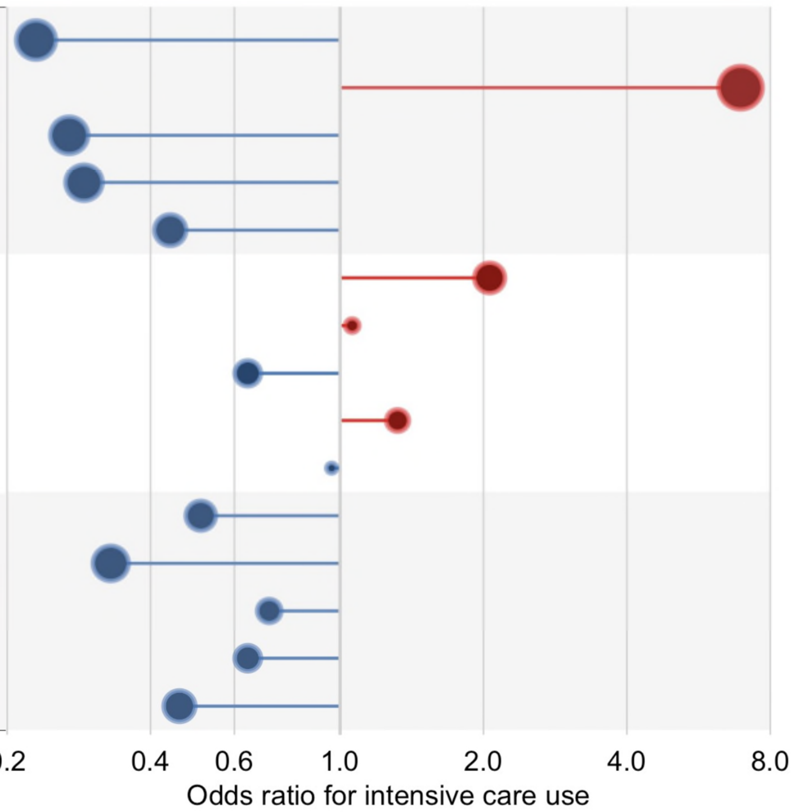

The adjusted odds ratio for the outcome was estimated per one unit increased in the eigen-value (the first principal component) of the corresponding module by fitting a multivariable logistic regression model with ridge regularization. 95% CIs were estimated by a bootstrap method with 2,000 replicates. In the model, we adjusted for age, sex, race/ethnicity, and respiratory virus. Statistically significant modules are in **bold**.

Abbreviations: BCAA, branched-chain amino acid; FDR, false discovery rate; GPCR, G-protein-coupled receptor; HR, host response; IFN, interferon; IL, interleukin; NADH, nicotinamide adenine dinucleotide hydrogen; MC, microbial composition

Supplemental Figure 6. Sensitivity analysis adjusting for age, sex, race/ethnicity, and virus: Integrated relationship of the dual-transcriptome modules with the risk of positive pressure ventilation use in infants hospitalized for bronchiolitis

Modules	Odds ratio (95% CI)
**T cell regulation module**	0.24 (0.11-0.53)
**Neutrophil/IL-1 module**	3.95 (1.71-10.1)
**GPCR module**	0.39 (0.16-0.78)
**Type I IFN module**	0.37 (0.14-0.75)
**HR-1 module**	0.43 (0.17-0.98)
***S.pneumoniae/S.aureus* module**	2.56 (1.19-5.81)
MC-1 module	1.68 (0.75-3.80)
Moraxella module	0.61 (0.24-1.46)
Streptcoccus module	1.72 (0.67-4.27)
Haemophilus module	1.44 (0.60-3.68)
**Plasma membrane module**	0.30 (0.16-0.50)
**mRNA metabolism module**	0.36 (0.20-0.64)
**BCAA metabolism module**	0.73 (0.05-0.88)
**Oxidative stress response module**	0.57 (0.07-0.78)
**NADH module**	0.59 (0.06-0.80)

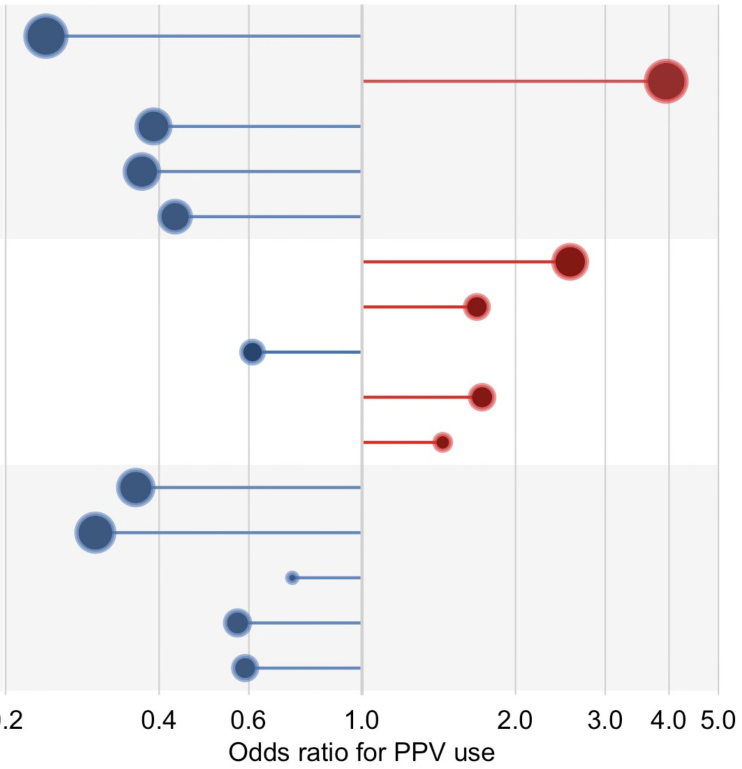

The adjusted odds ratio for the outcome was estimated per one unit increased in the eigen-value (the first principal component) of the corresponding module by fitting a multivariable logistic regression model with ridge regularization. In the model, we adjusted for age, sex, and respiratory virus. 95% CIs were estimated by a bootstrap method with 2,000 replicates. Statistically significant modules are in **bold**.
Abbreviations: BCAA, branched-chain amino acid; FDR, false discovery rate; GPCR, G-protein-coupled receptor; HR, host response; IFN, interferon; IL, interleukin; NADH, nicotinamide adenine dinucleotide hydrogen; MC, microbial composition

Supplemental Figure 7. Sensitivity analysis adjusting for age, sex, race/ethnicity, and virus: Integrated relationship of the dual-transcriptome modules with the risk of intensive care use in infants hospitalized for bronchiolitis

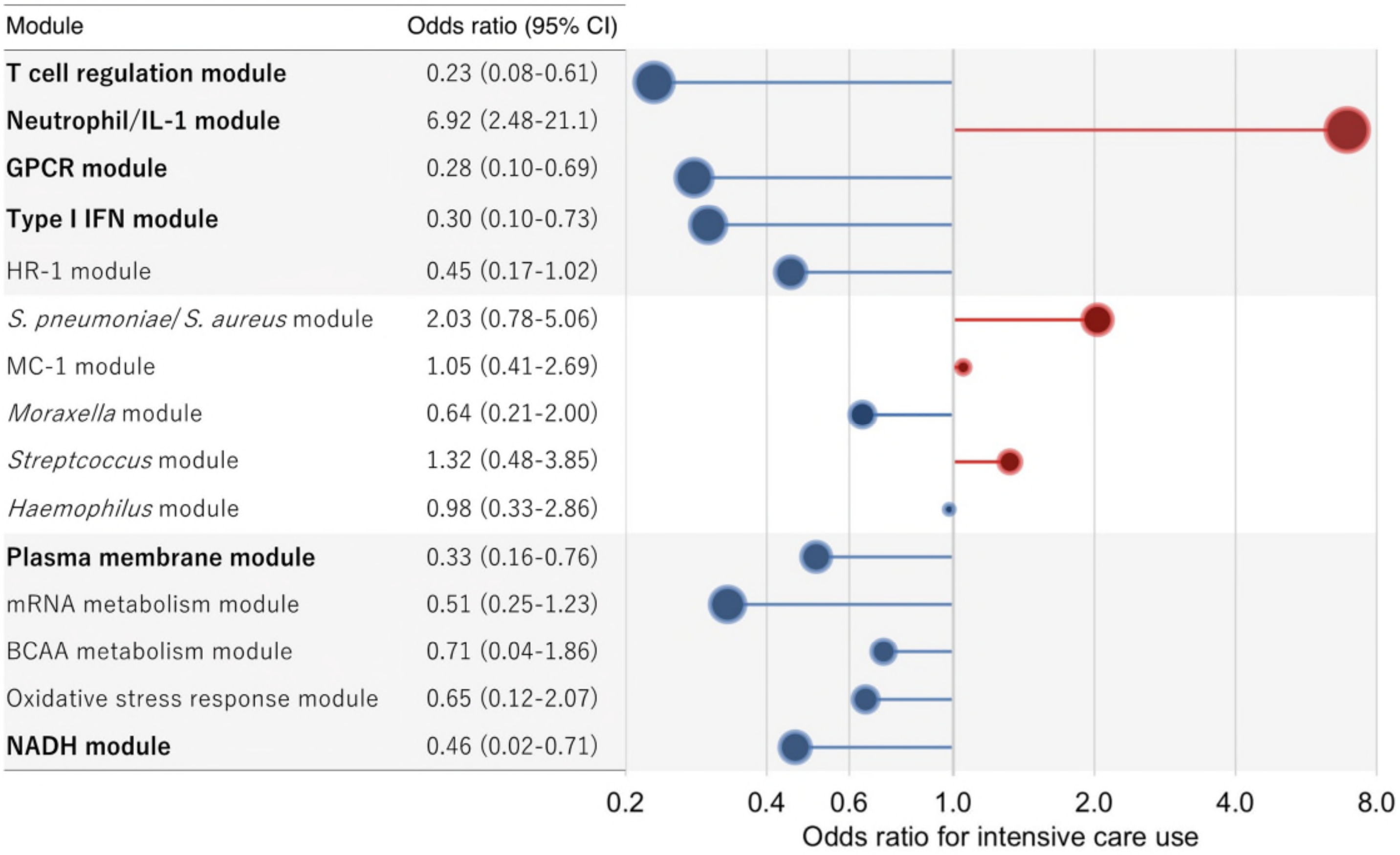

The adjusted odds ratio for the outcome was estimated per one unit increased in the eigen-value (the first principal component) of the corresponding module by fitting a multivariable logistic regression model with ridge regularization. In the model, we adjusted for age, sex, race/ethnicity, and respiratory virus. 95% CIs were estimated by a bootstrap method with 2,000 replicates. Statistically significant modules are in **bold**.
Abbreviations: BCAA, branched-chain amino acid; FDR, false discovery rate; GPCR, G-protein-coupled receptor; HR, host response; IFN, interferon; IL, interleukin; NADH, nicotinamide adenine dinucleotide hydrogen; MC, microbial composition

Supplemental Figure 8. Correlation network of major clinical variables and dual-transcriptome modules

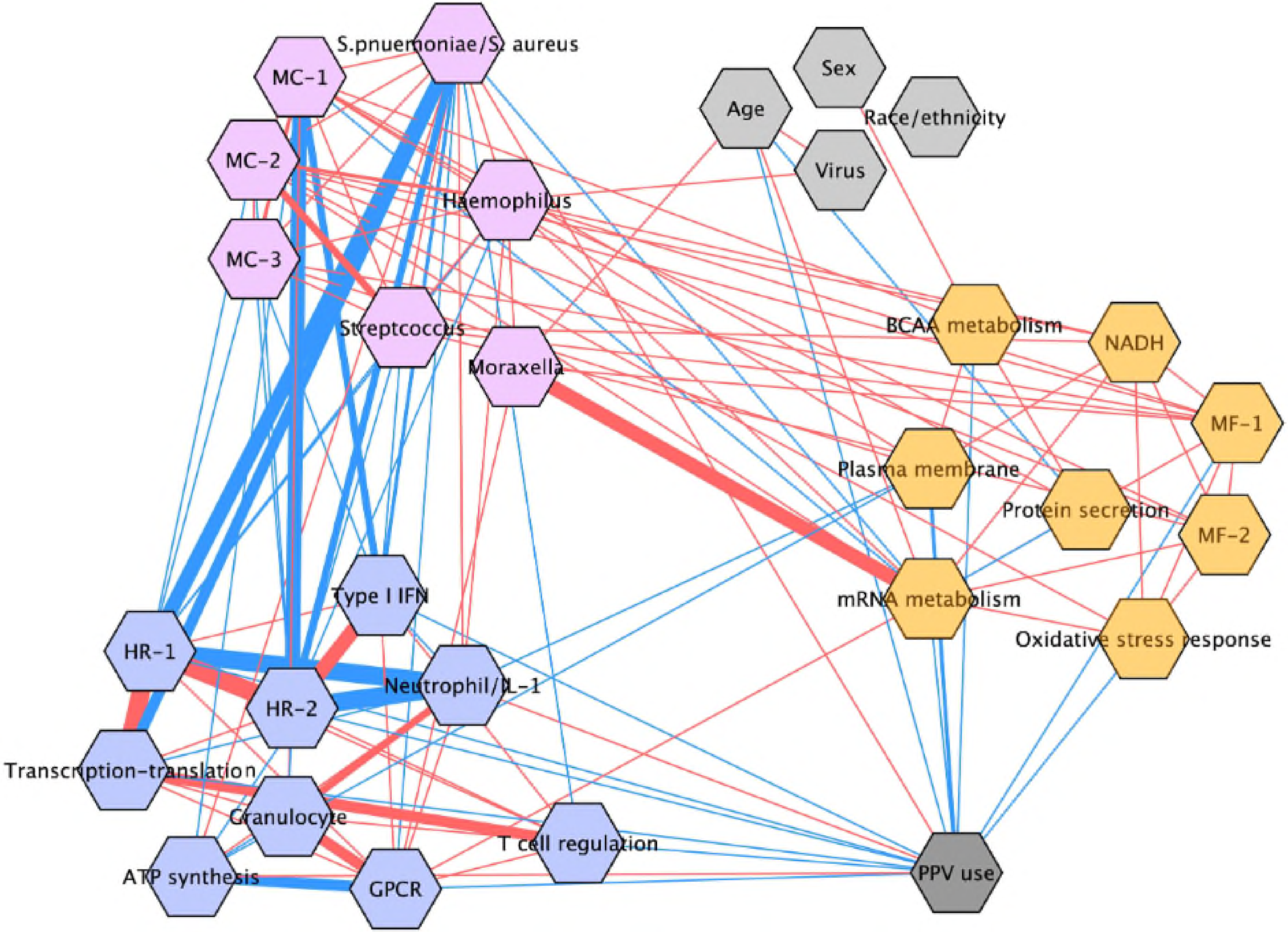

Nodes are represented by different colors corresponding to clinical variables and module categories. Edges show correlations between two variables and/or nodes. Edges with a Pearson correlation of greater than 0.15 are shown. Positive correlations are displayed as red; negative correlations are displayed as blue. Edge thickness is proportional to the strength of the correlation.

Abbreviations: ATP, adenosine triphosphate; BCAA, branched-chain amino acid; FDR, false discovery rate; GPCR, G-protein-coupled receptor; HR, host response; IFN, interferon; IL, interleukin; NADH, nicotinamide adenine dinucleotide hydrogen; MC, microbial composition; MF, microbial function

Supplemental Figure 9. Log-log plot of whole-network connectivity distribution of host response, microbial composition, and microbial function

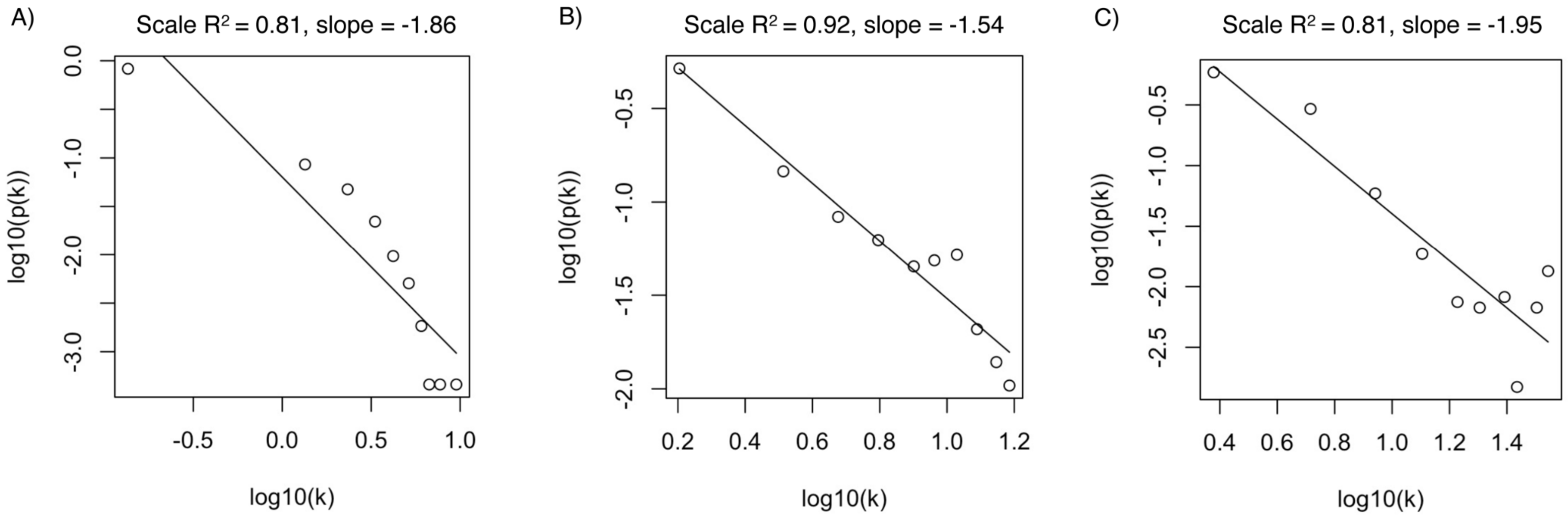

The x-axis indicates the logarithm of whole network connectivity; the y-axis indicates the logarithm of the corresponding frequency. On these plots, the distribution approximately follows a straight line, which implies an approximately scale-free topology in each dataset.

A) Host response data
B) Microbial composition data
C) Microbial function data
