# Supplementary material for: Integrated relationship of nasopharyngeal airway host response and microbiome associates with bronchiolitis severity
Source: Nat Commun. 2022 Aug 30;13:4970. doi: 10.1038/s41467-022-32323-y (PMC9427849; doi:10.1038/s41467-022-32323-y)
Supplement: Supplementary file 3 — Reporting Summary [file 41467_2022_32323_MOESM3_ESM.pdf]

nature portfolio

Corresponding author(s): Michimasa Fujiogi

Last updated by author(s): 2022/05/29

Reporting Summary

Nature Portfolio wishes to improve the reproducibility of the work that we publish. This form provides structure for consistency and transparency in reporting. For further information on Nature Portfolio policies, see our Editorial Policies and the Editorial Policy Checklist.

Statistics

For all statistical analyses, confirm that the following items are present in the figure legend, table legend, main text, or Methods section.

n/a	Confirmed	
☐	☒	The exact sample size (*n*) for each experimental group/condition, given as a discrete number and unit of measurement
☐	☒	A statement on whether measurements were taken from distinct samples or whether the same sample was measured repeatedly
☐	☒	The statistical test(s) used AND whether they are one- or two-sided<br>*Only common tests should be described solely by name; describe more complex techniques in the Methods section.*
☐	☒	A description of all covariates tested
☐	☒	A description of any assumptions or corrections, such as tests of normality and adjustment for multiple comparisons
☐	☒	A full description of the statistical parameters including central tendency (e.g. means) or other basic estimates (e.g. regression coefficient) AND variation (e.g. standard deviation) or associated estimates of uncertainty (e.g. confidence intervals)
☐	☒	For null hypothesis testing, the test statistic (e.g. *F*, *t*, *r*) with confidence intervals, effect sizes, degrees of freedom and *P* value noted<br>*Give P values as exact values whenever suitable.*
☒	☐	For Bayesian analysis, information on the choice of priors and Markov chain Monte Carlo settings
☒	☐	For hierarchical and complex designs, identification of the appropriate level for tests and full reporting of outcomes
☐	☒	Estimates of effect sizes (e.g. Cohen's *d*, Pearson's *r*), indicating how they were calculated

Our web collection on statistics for biologists contains articles on many of the points above.

Software and code

Policy information about availability of computer code

Data collection	REDCap: v10.0.30
Data analysis	R version 3.6.1<br>R package<br>-clusterProfiler: version 3.18.1<br>-fgsea: version 1.16.0<br>-wgcna: version 1.70-3<br>-glmnet: version 4.1-2<br>-caret: version 6.0-89<br>-pcalg: version 2.7-3<br>PathoScope (v2.0) used in this study can be found in the following site: https://github.com/PathoScope/PathoScope<br>Salmon (v1.4.0) used in this study can be found in the following site: https://combine-lab.github.io/salmon/<br>QUICS is a software owned by Metabolon<br>Cytoscape 3.9.1 used in this study can be found in the following site: https://cytoscpe.org/<br><br>Code availability: Computational code from the study is available at https://zenodo.org/badge/latestdoi/468931590.

For manuscripts utilizing custom algorithms or software that are central to the research but not yet described in published literature, software must be made available to editors and reviewers. We strongly encourage code deposition in a community repository (e.g. GitHub). See the Nature Portfolio guidelines for submitting code & software for further information.

Data

Policy information about availability of data

All manuscripts must include a data availability statement. This statement should provide the following information, where applicable:

- Accession codes, unique identifiers, or web links for publicly available datasets
- A description of any restrictions on data availability
- For clinical datasets or third party data, please ensure that the statement adheres to our policy

Data availability: The data that support the findings of this study are available on the NIH/NIAID ImmPort (https://www.immport.org/shared/study/SDY1883) through controlled access to be compliant with the informed consent forms of MARC-35 study and the genomic data sharing plan. Source data without participant-level data are provided with this paper.

KneadData v0.10.057
expanded Human Oral Microbiome Database (eHOMD) database
maxikraken2_1903 database (https://lomanlab.github.io/mockcommunity/mc_databases.html)
Bowtie2
EggNOG-mapper

Field-specific reporting

Please select the one below that is the best fit for your research. If you are not sure, read the appropriate sections before making your selection.

- [x] Life sciences
- [ ] Behavioural & social sciences
- [ ] Ecological, evolutionary & environmental sciences

For a reference copy of the document with all sections, see nature.com/documents/nr-reporting-summary-flat.pdf

Life sciences study design

All studies must disclose on these points even when the disclosure is negative.

Sample size	This is a secondary analysis of data from an observational study and the sample size of the final analytic cohort is 244. In this secondary analysis, a sample size calculation is not applicable. We have described the sample flow chart in the Supplementary Figure 1.
Data exclusions	The original cohort consisted of 1016 infants. Of these, the transcriptome and meta-transcriptome data were obtained in 244 infants who were randomly-selected from the cohort, contributing to the analytic cohort.
Replication	We confirmed reproducibility of the experimental findings using same code and dataset. However, we did not perform external validation. This is the first study that has showed complex interplay between host response, microbial composition, and its function, and their integrated relationship with the disease severity.<br>This study should facilitate further validation research.
Randomization	Not applicable. This is not a randomized trial. Besides, we do not have a "control" group. In this study, we have analyzed 244 infants with severe bronchiolitis.
Blinding	This is an observational study, so blinding is not applicable.

Reporting for specific materials, systems and methods

We require information from authors about some types of materials, experimental systems and methods used in many studies. Here, indicate whether each material, system or method listed is relevant to your study. If you are not sure if a list item applies to your research, read the appropriate section before selecting a response.

Materials & experimental systems

n/a	Involved in the study	
x		Antibodies
x		Eukaryotic cell lines
x		Palaeontology and archaeology
x		Animals and other organisms
	x	Human research participants
x		Clinical data
x		Dual use research of concern

Methods

n/a	Involved in the study	
x		ChIP-seq
x		Flow cytometry
x		MRI-based neuroimaging

Human research participants

Policy information about studies involving human research participants

Population characteristics	We enrolled infants (age <1 year) hospitalized with bronchiolitis at 17 sites across 14 U.S. states using a standardized protocol during three consecutive bronchiolitis seasons (from November 1 through April 30) during 2011–2014. The diagnosis of bronchiolitis was made according to the American Academy of Pediatrics bronchiolitis guidelines, defined as the acute respiratory illness with a combination of rhinitis, cough, tachypnoea, wheezing, crackles, or retraction54. We excluded infants with a pre-existing heart and lung disease, immunodeficiency, immunosuppression, or gestational age of <32 weeks, history of previous bronchiolitis hospitalization, or those who were transferred to a participating hospital >24 hours after initial hospitalization.<br>Of 1,016 infants enrolled into the cohort, the current analysis investigated 244 infants who were randomly selected for the dual-transcriptome profiling. The analytic and non-analytic cohorts did not significantly differ in the baseline characteristics , except for daycare use and RSV infection.<br>Among the analytic cohort, the median age was 3 (IQR, 2-6) months, 40% were female, and 42% were non-Hispanic white . Overall, 91% of study participants had RSV infection, 21% had rhinovirus (RV) infection, and 12% had RSV/RV coinfection. During hospitalizations for bronchiolitis, 7% of participants underwent PPV and 17% received intensive care treatment (defined by PPV use and/or admission to the intensive care unit).
Recruitment	Each morning from November 1 until April 30 in year 1 (2011-2012), and year 2 (2012-2013), a member of the site research team screened all children admitted with bronchiolitis to the medical ward, any "intermediate care" type of unit, and the intensive care unit, in the past 24 hours. Using a variety of mechanisms (e.g. patient logs, communication with medical teams, computerized registry, on-site research assistants), site investigators recorded whether they were either approached, missed (no attempt to approach), or known to be ineligible from pre-screen of record in the screening form. We enrolled children, male and female, of all races.<br>Once the general inclusion criteria (age <1 year, admitted to hospital, physician diagnosis of bronchiolitis, parent/legal guardian's ability to give informed consent) have been confirmed, study personnel approached the parent/legal guardian, related a brief overview of the study and asked if he or she is interested in hearing more about the study. If interested, study personnel administered the main section of the screening form to finally confirm eligibility. If the parent/legal guardian indicated a willingness to participate, the site investigator or study personnel obtained parent/legal guardian consent.
Ethics oversight	The institutional review board at each of the participating hospitals (see below) approved the study. Written informed consent was obtained from the parent or guardian.<br><br>Participating hospitals in MARC-35 are the following:<br>Alfred I. duPont Hospital for Children, Wilmington, DE<br>Arnold Palmer Hospital for Children, Orlando, FL<br>Boston Children's Hospital, Boston, MA<br>Children's Hospital of Los Angeles, Los Angeles, CA<br>Children's Hospital of Philadelphia, Philadelphia, PA<br>Children's Hospital of Pittsburgh, Pittsburgh, PA<br>The Children's Hospital at St. Francis, Tulsa, OK<br>The Children's Mercy Hospital & Clinics, Kansas City, MO<br>Children's National Medical Center, Washington, D.C.<br>Cincinnati Children's Hospital and Medical Center, Cincinnati, OH<br>Connecticut Children's Medical Center, Hartford, CT<br>Dell Children's Medical Center of Central Texas, Austin, TX<br>Norton Children's Hospital, Louisville, KY<br>Massachusetts General Hospital, Boston, MA<br>Phoenix Children's Hospital, Phoenix, AZ<br>Seattle Children's Hospital, Seattle, WA<br>Texas Children's Hospital, Houston, TX

Note that full information on the approval of the study protocol must also be provided in the manuscript.
